# A scalable, secure, and interoperable platform for deep data-driven health management

**DOI:** 10.1038/s41467-021-26040-1

**Published:** 2021-10-01

**Authors:** Amir Bahmani, Arash Alavi, Thore Buergel, Sushil Upadhyayula, Qiwen Wang, Srinath Krishna Ananthakrishnan, Amir Alavi, Diego Celis, Dan Gillespie, Gregory Young, Ziye Xing, Minh Hoang Huynh Nguyen, Audrey Haque, Ankit Mathur, Josh Payne, Ghazal Mazaheri, Jason Kenichi Li, Pramod Kotipalli, Lisa Liao, Rajat Bhasin, Kexin Cha, Benjamin Rolnik, Alessandra Celli, Orit Dagan-Rosenfeld, Emily Higgs, Wenyu Zhou, Camille Lauren Berry, Katherine Grace Van Winkle, Kévin Contrepois, Utsab Ray, Keith Bettinger, Somalee Datta, Xiao Li, Michael P. Snyder

**Affiliations:** 1 Department of Genetics, Stanford University, Stanford, CA USA; 2 Stanford Center for Genomics and Personalized Medicine, Stanford University, Stanford, CA USA; 3 Stanford Healthcare Innovation Lab, Stanford University, Stanford, CA USA; 4 Department of Computer Science, Stanford University, Stanford, CA USA; 5 Technology and Digital Solutions, Stanford Medicine, Stanford, CA USA; 6 Department of Biochemistry, The Center for RNA Science and Therapeutics, Department of Computer and Data Sciences, Case Western Reserve University, Cleveland, OH USA

**Keywords:** Computational platforms and environments, Data acquisition, Data integration

## Abstract

The large amount of biomedical data derived from wearable sensors, electronic health records, and molecular profiling (e.g., genomics data) is rapidly transforming our healthcare systems. The increasing scale and scope of biomedical data not only is generating enormous opportunities for improving health outcomes but also raises new challenges ranging from data acquisition and storage to data analysis and utilization. To meet these challenges, we developed the Personal Health Dashboard (PHD), which utilizes state-of-the-art security and scalability technologies to provide an end-to-end solution for big biomedical data analytics. The PHD platform is an open-source software framework that can be easily configured and deployed to any big data health project to store, organize, and process complex biomedical data sets, support real-time data analysis at both the individual level and the cohort level, and ensure participant privacy at every step. In addition to presenting the system, we illustrate the use of the PHD framework for large-scale applications in emerging multi-omics disease studies, such as collecting and visualization of diverse data types (wearable, clinical, omics) at a personal level, investigation of insulin resistance, and an infrastructure for the detection of presymptomatic COVID-19.

## Abstract (web summary)

The increasing scale and scope of biomedical data is generating tremendous opportunities for improving health outcomes, but also raises new challenges ranging from data acquisition and storage to data analysis and utilization. To meet these challenges, the authors develop the Personal Health Dashboard, which provides an end-to-end solution for deep biomedical data analytics.

## Introduction

Biomedical data continue to grow in scale, diversity, and complexity even as the costs of data access, storage, distribution, and analysis undergo rapid change^1–5^. These changes present researchers and clinicians with three major challenges: (1) Scalability: The majority of healthcare applications need to handle a large number of participants and different data types and tasks while also preserving rapid, actionable turnaround times (e.g., cardiovascular monitoring or infectious disease detection). At the same time, to allow for affordable precision medicine, researchers and physicians need cost-efficient systems that are able to scale dynamically. (2) Security: As the volume and diversity of data grow, the risk of violating privacy and security regulations increases. Researchers need systems that proactively mitigate re-identification risks by employing robust de-identification and end-to-end encryption methods. At the same time, these systems must also support strong logging, active monitoring, and auditing to preserve the privacy and security of participants by identifying and remediating issues before threats occur. (3) Interoperability: Vendor-specific technology products and services can be difficult and costly to integrate with other commercial products^6^. Researchers must be able to readily collect, store, and process large-scale biomedical datasets across multiple computing and storage platforms that are interoperable and low cost.

To address these three challenges, we developed the Personal Health Dashboard (PHD) platform, a secure, scalable, and interoperable platform that enables the streamlined and cost-effective acquisition, storage, and analysis of large biomedical datasets ranging from wearable biosensor data and multi-omics profiles to clinical data. PHD can operate on any large-scale cloud infrastructure or local high-performance computing (HPC) system and can be customized to a wide variety of end-user needs. We show how PHD can be used for collection and visualization of diverse datasets (wearable, clinical, omics) at a personal level, as an infrastructure for detection of presymptomatic COVID-19 cases, and biological characterization of insulin-resistance heterogeneity. It can support basic and clinical research studies, sophisticated analyses, and user interfaces. As such, we expect it to be of wide utility.

## Results

**Fig. 1:**
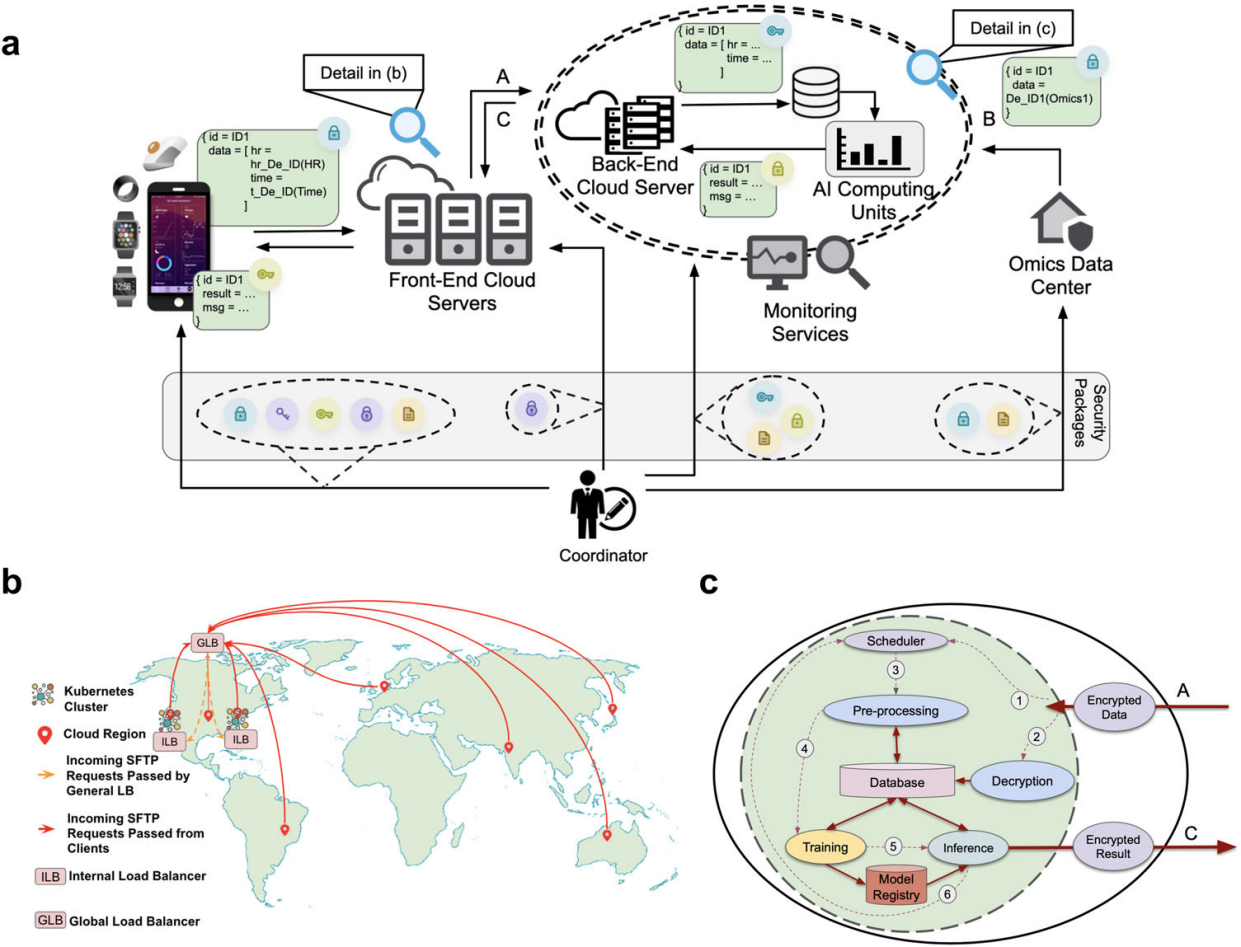
PHD Overview. **a** An overview of the PHD platform. The main components of the system are (1) Smartphone applications and wearable devices; (2) front-end authentication cluster; (3) back-end decryption cluster; (4) back-end ML cluster; (5) omics data center; (6) coordinator and security packages; and (7) network connections between different components. **b** Front-end, multi-regional, multi-zone cluster for authentication. In addition to a global latency-sensitive load balancer, every region of the cluster has its own load balancer that helps the region to scale independently. We configured SFTP on the Kubernetes cluster using SFTPGo^50^, which maps each user to a bucket virtual folder. **c** An overview of the back-end ML cluster. The data flow is as follows: (1) New incoming data from the authentication cluster triggers the scheduler. (2) The decryption cluster decrypts the new data and pushes it into the distributed database. (3) The scheduler triggers a ML pipeline starting with a preprocessing stage. (4) Preprocessing pushes the preprocessed data into the database and triggers the corresponding training stage. (5) Updated model gets pushed into the model registry by the training stage and triggers the corresponding inference stage. (6) Inference stage pulls the updated models, runs the corresponding inference, encrypts the output, and sends it to the authentication cluster. Depending on the input application, Steps 4–6 can be combined.

The PHD platform is designed to provide researchers with a secure, scalable, and easy-to-use end-to-end system for conducting wearables and multi-omics biomedical data research studies, including capture, visualization, and integration of diverse datasets. An overview of key PHD components is provided in Fig. 1a. The platform facilitates biomedical and wearable data collection, including interaction with study participants via a dedicated PHD smartphone app. User data are de-identified on the smartphone at the earliest stage and transferred to the PHD back-end through a front-end authentication cluster. The PHD back-end consists of a decryption cluster and a machine learning (ML) cluster. Through secure integration of omics data centers, the PHD platform allows for joint analysis of wearables data with multi-omics and clinical data on its ML cluster.

The work and data flow of the platform are organized as follows (Fig. 1a): The first stage is user registration, during which the coordinator creates a new user by generating the required credentials, IDs, and attributes, such as username, password, data de-identification values, IDs, data encryption public key, analysis result decryption private key, SFTP public/private key pairs, etc., and sends the required matching credentials to the user. It also sends the required info, including the SFTP public key, to the front-end authentication cluster to register for end-to-end encryption. Also, the data decryption private key, analysis result encryption public key, and data de-identification values are sent to the back-end decryption cluster, and finally, the data encryption public keys and IDs are sent to the omics data center (e.g., the iPOP data repository^7^), where the omics data center de-identifies and encrypts multi-omics data, and then transfers the data to the back-end ML cluster, where omics data can be analyzed jointly with collected wearables data.

### PHD is a scalable platform for data storage and analysis

Each component of the PHD platform was designed to be scalable to make importing, querying, and analyzing large medical databases feasible at high speed and low cost. To achieve scalability and security in the front-end authentication cluster, we leveraged Kubernetes^8^ and the SSH File Transfer Protocol (SFTP) which enables end-to-end encrypted data transmission at scale. To increase availability and fault-tolerance, as well as to ensure rapid response time, we configured a multi-regional and multi-zone cluster using Terraform (an open-source tool for building, changing, and versioning infrastructure)^9^ (Fig. 1). The PHD smartphone app de-identifies and encrypts data at the earliest possible stage. After transferring the de-identified and encrypted data through the authentication cluster, the data is then transferred to a secure computing environment (as visualized by the green circle, see Fig. 1c) which has the highest level of security and monitoring. This secure computing environment comprises two major components: decryption and ML clusters for data processing and biomedical research, respectively.

To achieve scalable decryption, the decryption cluster uses an event-driven, serverless architecture (e.g., AWS Lambda^10^, Google Cloud Functions^11^, Apache OpenWhisk^12^, and Azure Functions^13^). Upon receiving new data, the decryption cluster triggers the scheduler of the ML cluster. Ideally, the ML cluster will process any new job immediately. However, the scheduler must prioritize processing tasks based on the urgency of the job and available resources. For example, a job for a real-time analysis (e.g., the Change-of-Heart algorithm presented in ref. ^1^) can be prioritized compared to a long-term activity project. There are three events that impact the scheduler: (1) incoming new data, (2) incoming jobs for analysis, and (3) chains of events, in which one analysis initiates another analysis (e.g., a blood sugar level analysis result potentially triggers a comprehensive diabetes analysis). After the scheduler triggers preprocessing and (potentially) training stages, the ML cluster uses MLflow^14^ to manage the ML lifecycle, including experimentation, reproducibility, deployment, and versioning in a central model registry.

**Fig. 2:**
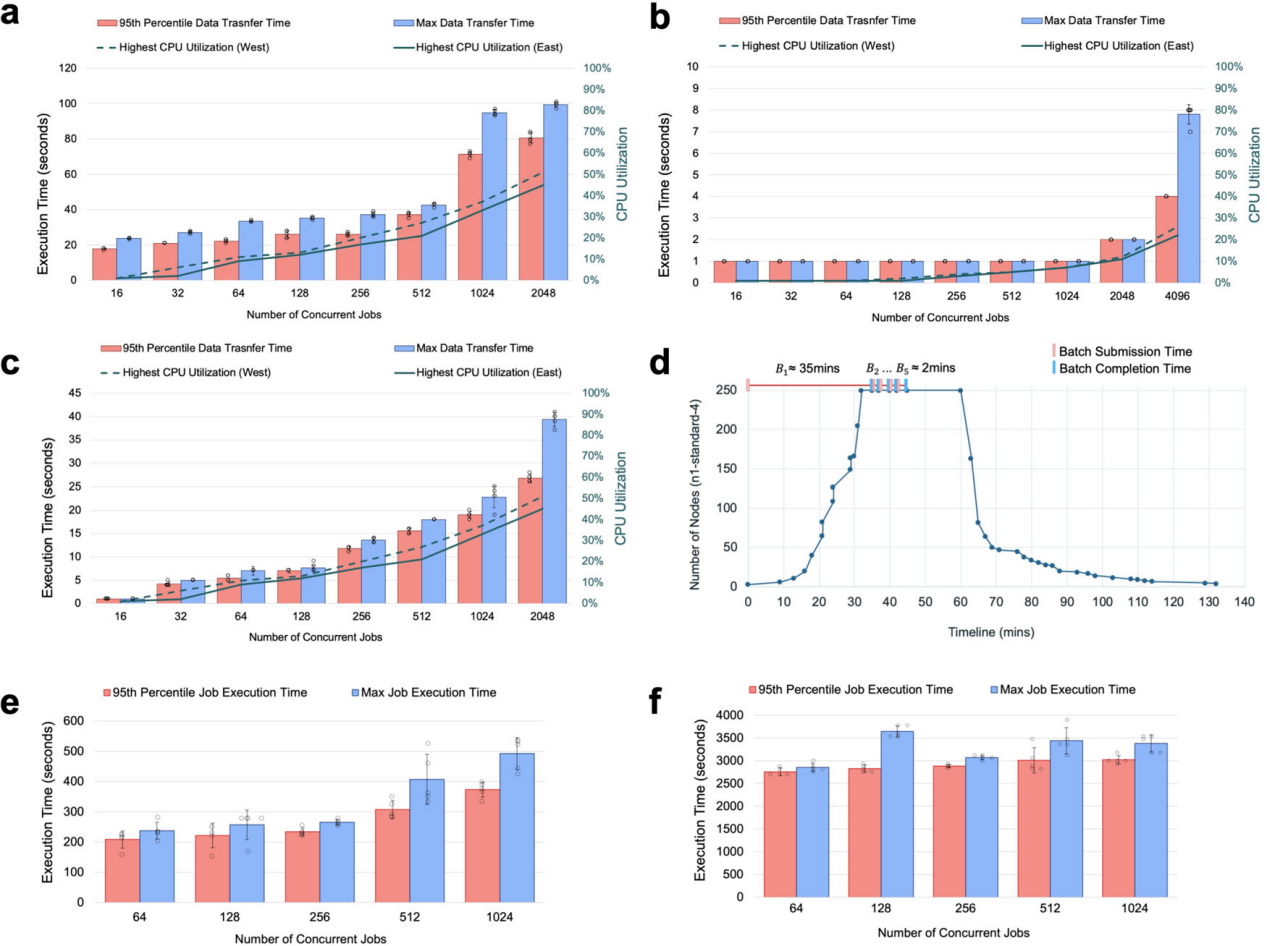
Scalability analysis of the PHD platform. In panel **a**, the workload size was 100MB (historical data) and the CPU utilization for the regional sub-clusters (i.e., east and west) significantly increased for batches with more than 512 concurrent jobs. In panel **b**, the workload size was 1 MB (real-time data), and the CPU utilization for the regional sub-clusters increased slightly even for 4096 concurrent jobs, with a maximum response time below 10 seconds. In panel **c**, the workload was a mixture of both historical and real-time jobs. In this figure, 20% of the jobs are 100 MB, and the rest are 1 MB. If we consider a 30-second response time as an acceptable threshold, then for 2048 concurrent jobs, the response time for the 95th percentile of jobs is still below 30 seconds while CPU utilization remains below 40%. Therefore, a good threshold for scaling up the cluster is when the CPU utilization is ≤40%. **d** In this experiment, five batches of 1000 jobs were submitted back to back, each job needing one vCPU. When we submitted the first batch of 1000 jobs, it took almost 35 minutes for Kubernetes to scale up from 3 nodes to 250 nodes of N1 machine type with 4 virtual CPUs (Google Cloud n1-standard-4) or 1000 vCPU cores, and the maximum response time was 35 minutes for batch 1 (B1). For batch 2 to batch 5 (B2, ..., B5), the cluster had enough computing resources and processed the entire batch of jobs in almost 2 minutes. Finally, after new jobs were no longer sent to the cluster, CPU utilization dropped, and it took 90 minutes for Kubernetes to scale down to 3 nodes. **e**, **f** These figures cover weak scaling experiments around the ML cluster using distributed messaging Pub/Sub and Kubernetes cluster. These figures indicate ML cluster scales well under weak scaling and keeps the execution time within the same range. **a**, **b**, **c**, **e**, **f** were run five times. Data points, average values and standard deviations were reported.

We classified the front-end workloads in PHD into two major types: (1) historical data (e.g., data collected in the past three months) and (2) real-time data (e.g., last hour). Figure 2a–c shows the performance of the authentication cluster under these workloads. For this set of experiments, we configured horizontal autoscaling on a multi-regional and multi-zone cluster with 18 nodes of N1 machine type with 8 virtual CPUs (Google Cloud n1-standard-8). The *x*-axis shows the number of concurrent jobs sent to the cluster from 8 regions reported in Methods subsection “Authentication cluster”, and the left *y*-axis and right *y*-axis represent the response time in seconds and the CPU utilization, respectively. Figure 2d shows how a Kubernetes cluster scales dynamically for an input burst of jobs. The workload included the calculation of a number of statistics for each day of user data (e.g., HR stratification, number of outliers). The cluster had a node autoscaling policy of 3 and 250 nodes as the minimum and maximum number of nodes, respectively, as well as a horizontal, autoscaling policy of 50% CPU utilization. The *x*-axis shows the timeline in minutes, and the *y*-axis shows the number of nodes. This experiment shows systems like Kubernetes with proper configuration can significantly decrease infrastructure costs. Figure 2e, f covers weak scaling experiments around the ML cluster using distributed messaging Pub/Sub and a Kubernetes cluster. Weak scaling typically involves scaling the problem size and the number of containers at the same rate such that the problem size per container is fixed. In Fig. 2e, we increased the number of preprocessing jobs (Step 4 in Fig. 1c), and the cluster scales accordingly. One limitation was the number of concurrent jobs in BigQuery. Figure 2f shows the performance of combined training and inference jobs (combined Steps 5 and 6 in Fig. 1c) under weak scaling. Each job first trains a model and then executes the inference section.

To ensure the back-end database of the PHD platform can be scaled dynamically based on the number of read and write jobs, we configured our system on distributed SQL query engines: BigQuery on GCP^15,16^, Athena on AWS^17^, and Apache Presto^18^. In our implementation, every participant has their own dataset and set of tables. Overall, these results demonstrate that PHD is compatible with the major distributed SQL databases.

### PHD provides a secure environment

The PHD platform applies several principles and technologies to ensure a secure big data environment. The first important privacy tenet is that no Protected Health Information (PHI) data are used (stored or transferred) throughout the platform. The second important factor is that all data in transit and at rest are end-to-end encrypted and de-identified using the best practices in cipher suites; hence, even if an attacker accesses any data, it would be encrypted and de-identified. In addition to these protections, (i) in the PHD mobile app, we follow the Open Web Application Security Project (OWASP) guidelines^19,20^, such as secure local user credential storage, prevention of running on rooted environments, code obfuscation, tamper detection, and additional features as described in Methods subsection “Security”; (ii) in the PHD communication channel, we leveraged SFTP and all data are encrypted using RSA asymmetric encryption with padding; and (iii) in the PHD authentication cluster, we deployed best practice authentication policies, such as login throttling for controlling brute-force attacks using Fail2ban^21^.

**Fig. 3:**
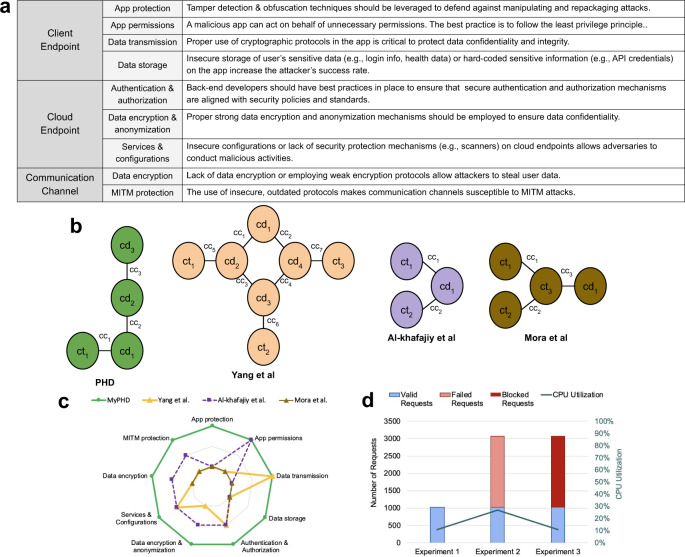
Security and privacy analysis of PHD platform. **a** Security and privacy best practices of each component of a healthcare platform. **b** Abstract graph model of PHD and three other similar healthcare platforms^51–53^. A healthcare platform is mapped to an abstract graph model as a set of vertices (V) and edges (E). Vertices can be client endpoints (ct) (wearables are not considered a separate vertex but instead part of a ct vertex), a set of cloud endpoints (cd), and the edges are communication channels (cc) used to transfer data from component to component. Yang et al.^51^ propose a wearable ECG monitoring system based on the internet-of-things (IoT) cloud. Al-Khafajiy et al.^52^ develop a smart healthcare monitoring system to accumulate elderly people’s physiological data via wearable sensors and transmit data to the Cloud for analysis and processing. Finally, Mora et al.^53^ propose a distributed framework based on the IoT paradigm for monitoring human biomedical signals during activities. **c** Security and privacy comparison of PHD and similar existing platforms using a component-based analysis. Each spoke has three data points: (1) not supported, (2) partially supported, and (3) fully supported. Missing data points represent cases in which evaluation was not available in existing works (e.g., data encryption and anonymization does not exist in Mora et al.). **d** Cost of login throttling authentication policy in PHD. The left *y*-axis and right *y*-axis represent the number of jobs and the CPU utilization, respectively. Note that the impact of fail2ban is insignificant: CPU utilization increased from 10.54% (without fail2ban) to 11.24% (with fail2ban).

To compare the security of PHD to other similar state-of-the-art platforms^22–26^, we deployed an abstract graph model that segments healthcare platforms into components/endpoints (via vertices) and represents how these components connect to each other (via edges). Using the abstract graph model, we then compared these platforms by examining the security and privacy properties of each component, as described in Fig. 3a. Figure 3b compares the abstract security graph model of PHD to three other similar healthcare platforms. As Fig. 3c depicts, we deployed best practices in security and privacy preservation, whereas other similar platforms fail to satisfy these best practices with respect to standard security guidelines. Finally, Fig. 3d shows the results of our brief evaluation of the impact of the authentication policy (Fail2ban) on the performance of the authentication cluster in terms of CPU utilization. Thus, PHD is able to run efficiently, even while highly secure.

### PHD provides interoperability

The entire PHD platform is deployable on any cloud provider and HPC system, which are listed in Supplementary Table 1. Details of the adaptation and configuration of Kubernetes clusters, serverless, event-based platforms, and back-end databases are provided in Methods subsection “Scalability”.

In addition to platform interoperability, PHD provides researchers with means for device-agnostic collection of real-time and historical wearables data as well as multi-domain datasets (e.g., clinical data, DNA sequencing, and other omics data processed files). To this end, PHD supports all common APIs such as OAuth 2.0 in case of Fitbit or the HealthKitAPI in case of Apple or Garmin Watches. SDKs such as the Tizen SDK in case of Samsung Galaxy Smartwatches are also supported. Data from any device is processed using PHD’s core data collection pipeline including data acquisition, harmonization, de-identification, and encryption on the smartphone level. In addition, PHD’s data collection APIs can be further customized based on the details inherent to each device.

In addition to platform interoperability, PHD provides researchers with means for device-agnostic collection of real-time and historical wearables data as well as multi-domain datasets (e.g., clinical data, DNA sequencing, and other omics data processed files). To this end, PHD supports all common APIs such as OAuth 2.0 in case of Fitbit or the HealthKitAPI in case of Apple or Garmin Watches. SDKs such as the Tizen SDK in case of Samsung Galaxy Smartwatches are also supported. Data from any device is processed using PHD’s core data collection pipeline including data acquisition, harmonization, de-identification, and encryption on the smartphone level. In addition, PHD’s data collection APIs can be further customized based on the details inherent to each device.

**Fig. 4:**
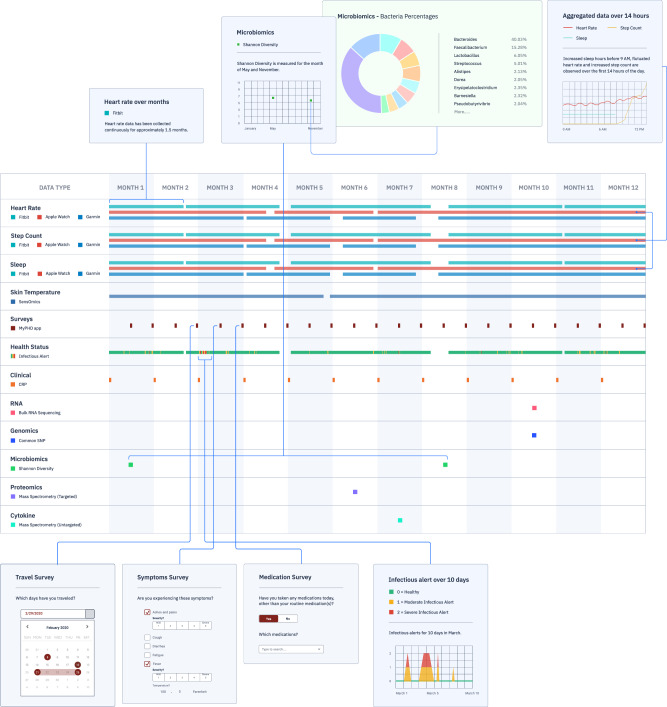
An illustration of longitudinal integration of wearables, surveys, and multi-omics data in PHD. Displayed is a schematic of a longitudinal record for a single user as generated in the PHD back-end. All measurements recorded via the PHD smartphone app, surveys, and wearables data include timestamps. The PHD de-identification process allows for preservation of relative times within a user record. Any multi-omics data added to the PHD platform via the multi-omics data center architecture can be mapped onto the user’s record based on its timestamp. The aggregated user record can then be preprocessed and analyzed in the ML cluster to the researchers will, allowing for joint analysis of wearables and multi-omics data in longitudinal format.

**Fig. 5:**
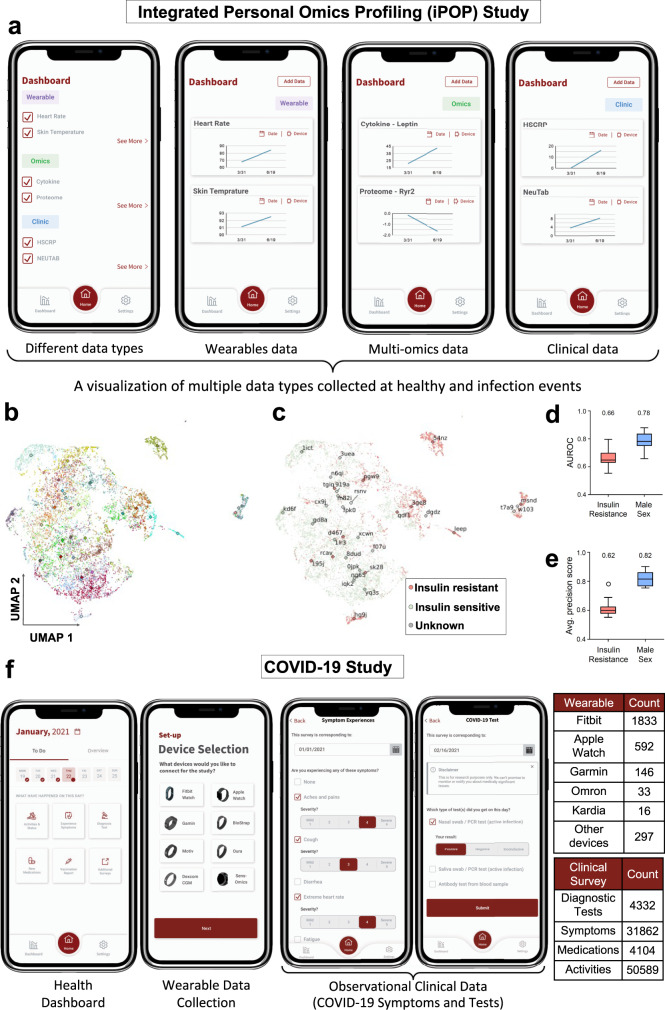
Real-world application studies of the PHD platform. **a** An example of using the PHD platform for data integration and visualization at an individual level; data collected through the iPOP study is displayed: a healthy event on 3/31 vs. viral infection event on 6/19. The PHD app provides an easily accessible interface (dashboard) for multiple data types, integration of surveys, and health-related predictions or information for real-time health monitoring for wearable, multi-omics, and clinical data. **b**–**e** An example of using the PHD platform for data analysis at a cohort level. **b** A UMAP^47^ visualization of the cohort’s wearables data. Colors represent individuals and differences in individual wearables data are visible. Each dot represents 24 h of wearables data. **c** Demonstrates the same feature space as in (**b**), but labeled by the insulin status of the respective individual. Red color represents insulin resistant, green color represents insulin-sensitive individuals, respectively, while individuals of unknown status are indicated in gray. Separation by disease status is visible. For panels **b** and **c**, the diamond symbol represents the median of an individual and random IDs (e.g., 54nz) are annotated. **d** A boxplot summarizing the distribution of area under the receiver operating characteristics (AUROC) of the logistic regression models trained on user-level, aggregated features for the binary classification problems of IR vs IS (insulin-resistant vs. insulin-sensitive) and male sex in a 10× 5-fold cross-validation. **e** Shows the distribution of average precision scores of the classification. In both plots, the distribution consists of *N* = 10 measurements of performance of the outer cross-validation loop. The box indicates the interquartile range (IQR), with the central horizontal line indicating the median. The whiskers extend to the last non-outlier data-point within 1.5*IQR. **f** An example of applying the PHD platform for a COVID-19 detection study^30^. Data from 2831 participants have been collected through the PHD platform. Summary of collected wearable data from various manufacturers, and clinical surveys are reported in the corresponding tables.

All data collected in the PHD platform via the PHD smartphone application are timestamped in an event-based approach (similar to other Common Data Models such as OMOP^27^). Therefore, collected wearables, multi-omics, and survey data can be aggregated in a longitudinal record format depending on the use case of the researcher. All data are mapped onto the user’s record based on their annotated timestamps (see Fig. 4). All structured wearable, clinical, and processed multi-omics data along with metadata were stored in GCP BigQuery, AWS Athena, and Apache Presto using the SQL standard which enables a high degree of portability and interoperability; unstructured data were stored on AWS S3 and Google Cloud Storage. The records can be queried depending on a researcher’s application and analyzed in the PHD ML cluster. Analysis results are then sent back to the mobile app on user devices for visualization, as exemplified in Fig. 5. The return of results is structured as needed by the application, such as real-time alerting of potential COVID infection.

In order to enable interoperability in mobile platforms, we implemented the PHD mobile app for the two most widespread platforms dominating the market: iOS and Android. For both platforms, we used the native mobile app development approach (Swift for iOS and Java for Android). The native mobile app development approach was specifically selected under privacy considerations–development in a web or hybrid approach is generally easier and faster but it poses more security engineering challenges. For instance, developers cannot leverage the native security and privacy best practices for web or hybrid apps, whereas they are extensively available for native apps.

The PHD app makes it possible to collect lifestyle- and health-related information surveys (e.g., travel information, symptoms, activities, medications, clinical test results). This flexible mobile framework enables more frequent and real-time data collection of health reports in an interactive manner.

### PHD supports data visualization for users and computationally intensive analytics

In this section, we present two case studies to demonstrate distinct features of the PHD framework. In the first study, the iPOP study (Stanford IRB #34907, #23602)^3,28^ presented in Fig. 5a, e, the PHD platform allows for joint analysis of wearables data with multi-omics and clinical data on its ML cluster through secure integration of omics data centers. Here, we collected longitudinal wearables, clinical, and multi-omics data from ~100 participants. The different data types (wearables, clinical, omics) can be displayed to the users at their preferred time scale (Fig. 5a).

We also performed a descriptive analysis of historical wearables data for personal comparison within a cohort as well as prediction of phenotypic traits such as insulin resistance (Fig. 5b–e). For each iPOP participant, we generated 12 domain-knowledge-based features, such as the average heart rate, sleep, and activity-based heart rate stratification or total step counts from the Fitbit smartwatch data for each day (Methods subsection “Extraction of domain-knowledge-based features”, Supplementary Table 2). Clustering of resulting feature space demonstrates that days from the same individuals tend to aggregate together as a personal baseline enabling personal comparison with other members of a cohort (Fig. 5b, c).

To test the potential use of this data for computational analysis in a medical context, we aggregated the extracted features per individual (see Methods subsection “Extraction of domain-knowledge-based features”) and trained a regularized logistic regression model to distinguish insulin-resistant from insulin-sensitive individuals as well as predicting their sex. Trained in a 10× 5-fold cross-validation setting, our models achieved a test-set area under the receiver operating characteristic (AUROC) of 0.78 and 0.66 with test-set average precision scores of 0.82 and 0.62 for predicting insulin resistance and male sex, respectively. All preprocessing steps from integration of clinical information to computation were conducted in the PHD back-end leveraging the PHD cloud infrastructure.

For the second study, in response to the COVID-19 pandemic, we rapidly adapted PHD to support early COVID-19 detection (Stanford IRB #55577^29^^,30^) via longitudinal wearable devices data. During a period of three months, the PHD platform successfully collected data from 2831 participants (Fig. 5f), and served as the foundation for multiple generations of COVID-19 detection algorithms capable of flagging signal abnormalities up to 10 days before symptoms manifested. The open-source PHD platform is designed to provide researchers with a secure, scalable, and easy-to-use end-to-end system for conducting real-time wearables studies where the participant wearable and longitudinal survey data can be combined with electronic health record and other data types. The secure cloud agnostic platform allows data capture, integration of diverse datasets, analytics, visualization, and finally, notification to patients via the native mobile PHD smartapp. The framework is now capable of sending alerting messages back to the user. Thus, these different examples demonstrate that PHD is a versatile system for collecting wearable and other data types, displaying them to users and performing analyses on these data as well as enabling real-time user interactions.

## Discussion

We introduced PHD to provide a secure, scalable and interoperable software platform that allows individuals, physicians, and researchers to collect store and interrogate large biomedical datasets to attain biomedical insights. This generic platform can be quickly adapted and configured on any large-scale cloud infrastructures or local high-performance computing (HPC) systems. It is readily applicable to two major types of data analyses: (1) Individual analysis, for instance, the utilization of wearable devices to detect physiological abnormality related to a certain disease (e.g., heart failure^31^, COVID-19 onset), which often requires real-time feedback to the individual to enable immediate clinical attention; (2) Cohort analysis, for instance, the integration of biomedical data to classify individuals into different groups, which requires researchers to access data from a group of participants. We showcased the capabilities of the PHD platform for both individual and cohort analyses. In the future, the highly secure nature of PHD has the potential to provide a valuable data-sharing platform for physicians and researchers reducing barriers between data silos. The PHD platform was prototyped on Google Cloud Platform. The platform is easily deployable on other cloud providers and HPC systems Supplementary Table 1. Major challenges inherent in the use of Kubernetes clusters, serverless, event-based platforms and back-end databases are reported in Methods subsections “Authentication cluster”, “Decryption cluster”, and “Back-end database”. Notably, the PHD platform does not contain any PHI data, thus preventing privacy breaches by design. However, integrating multi-omics data into the PHD system could introduce new privacy challenges (e.g., genomics data) as described in Methods subsection “Data anonymization”. Other frameworks supporting research studies and data models, including mobile and wearable devices, have been previously reported^32–34^. While these frameworks generally support participant integration and data collection via dedicated apps, they differ from PHD in two important aspects. First, these frameworks do not support data analysis in the same compute environment, requiring the setup of costly resources or data transfer to respective compute sites. Second, unlike existing systems that store all data (including PHI data) on public cloud providers, PHD provides meaningful results at scale by running large-scale ML studies using only de-identified data. PHD achieved this by balancing security and scalability via a clear separation of PHI and non-PHI information and implementing the event-based model end-to-end to support various data types. The PHD platform overcomes myriad long-standing challenges in integrating wearable, multi-omics, and clinical data within one general computing framework, thereby allowing secure collection, integration, and analysis of heterogeneous patient data for large-scale longitudinal studies. Thus, our platform can foster and facilitate biomedical research and promote precision medicine and personalized prevention.

## Methods

### Security

#### Local data storage

In the PHD app (available on both Google Play^35^ and App Store^36^—upon acceptance, we will release the source code of the app), we do not store any user data locally. The only data stored on the app is the user’s credentials. We securely store all credentials in the KeyStore (Android) and KeyChain (iOS), as it is recommended by OWASP^19,20^. To block adversaries from accessing the credentials even with root privileges on the device, PHD stores all credentials in encrypted form. To prevent illegitimate use on jailbroken/rooted devices, we also tested the PHD app in a rooted environment where we ensured all data are securely encrypted using KeyStore/KeyChain tools and resulting in no plain-text local data storage.

#### Reverse engineering defense

In addition to secure credentials storage, the PHD app leverages several reverse engineering defense and root/tamper detection mechanisms. For root detection, first, we check for several signs that commonly appear in rooted environments such as “su” binaries and package files of common rooting apps. Besides this, we implemented SafetyNet^37^ in the PHD app as an additional in-depth defense signal as part of an anti-abuse system as suggested by Google. For tamper detection, in multiple places of the app, the app verifies the signing certificate at runtime to ensure the certificate remains consistent; with this method, we can detect any app tampering and terminate upon any code injection.

#### Mobile app security evaluation

We evaluated the PHD app security via Mobile Security Framework (MobSF)^38^, a popular penetration-testing, malware analysis, and security assessment framework to ensure all security and privacy requirements suggested by OWASP are implemented.

#### Data anonymization

Preserving user privacy in the domain of multi-omics data has not been widely studied^39,40^. Humbert et al. have shown practical de-anonymization attacks on genomics data are feasible even in a relatively small dataset^41^. We use the following anonymization approach: (1) we anonymize the keys (i.e., date-time) by anchoring them to arbitrary different points in the future for each participant while preserving the time-series property and (2) we anonymize the values (e.g., heart rate) by adding a slight amount of noise such that the utility (same results from analysis algorithms) is preserved. Given the lack of comprehensive research on security and privacy risks in omics data, our future work will focus on algorithms capable of simultaneously delivering high privacy protection (anonymization) and utility preservation while integrating various omics data, such as metabolomics, lipidomics, and proteomics, into the PHD platform for biomedical research.

#### HIPAA compliance best practices

Even though we do not store any PHI data in the PHD platform, we have nonetheless leveraged security, privacy, and compliance best practice controls recommended for healthcare data in a HIPAA-aligned project (e.g., setting up *owners*—with multi-layer access control protections—and *auditors* groups, establishing *audit* logs that track access to all services, and creating *Forseti* instances that detect cloud configuration anomalies)^42^.

### Scalability

#### Authentication cluster

The authentication cluster is configured in two regions (us-west-1 (Oregon) and us-east-1 (South Carolina)), each region having three zones and each zone having three nodes. We submitted different workloads as concurrent requests to the cluster from eight regions (us-central1-a (Iowa), us-west1-b (Oregon), us-east4-c (Northern Virginia), southamerica-east1-b (Sao Paulo), australia-southeast1-b (Sydney), europe-west3-c (Frankfurt), asia-south1-c (Mumbai), and asia-northeast2-a (Osaka)), as tagged in Fig. 2. We considered a 30-s response time an acceptable threshold and for 2048 concurrent requests, the response time for the 95th percentile of requests was still below 30 s with CPU utilization below 40%. From this, a good threshold for scaling up the cluster was ≤40% CPU utilization.

Kubernetes has three levels of autoscaling: (1) a Horizontal Pod Autoscaler (HPA) that controls the number of replicas in a deployment, (2) a Vertical Pod Autoscaler (VPA) that controls the amount of requested resources (CPU and memory) for a pod, and (3) a Cluster Autoscaler that controls the number of nodes in a cluster. To achieve scalable and cost-effective solutions, system engineers will need to study application behavior and workloads by collecting detailed information with the help of profiling and tracing toolsets to configure these autoscaling mechanisms.

#### Decryption cluster

From start to end, the decryption process is triggered when encrypted data are sent to the authentication cluster by the user and ends when the user’s data are decrypted and appended to our access-controlled back-end database (e.g., Google Cloud BigQuery). Upon new data arrival, an event is triggered via a serverless, event-based platform (e.g., Google Cloud Function). Once an event is triggered, the decryption function is called, which first verifies the file and then fetches the separately-stored decryption keys associated with the user ID and decrypts the data block by block and appends it to the back-end database.

It is important to consider the maximum function duration (e.g., the timeout is 540 s in GCP Cloud Functions^43^ and 900 s in AWS Lambda^44^). To overcome this limitation, we partitioned files into chunks that were handled before function timeout.

#### Terraform

The infrastructure backing the project is managed using the open-source tool Terraform^9^ which allows the configuration of software deployments to be declaratively defined in their entirety. Representing the infrastructure in Terraform’s deterministic format allows changes to be audited as part of code review and provides self-healing capabilities. Significant flexibility is introduced by parameterizing the deployment such that it can scale to any level of throughput demanded. This includes being able to provision new cloud regions with a simple configuration change.

#### ML cluster

Workload heterogeneity demands different computing resources (e.g., preprocessing stages with or without GPU resources) that should scale independently. Therefore, to efficiently address this challenge, we created an internal Kubernetes cluster with a set of node pools (i.e., a group of nodes within a cluster that all have the same configuration). We also bundled tasks with similar computing needs as a pod (i.e., the basic execution unit of a Kubernetes application), and associated different pods to different node pools. Every node pool has its own internal load balancer, and it scales independently. With this design, we were able to handle real-time data analysis at both the individual level and the cohort level.

We considered two major designs for the implementation of the machine learning (ML) cluster: (1) Model Registry, a single repository that provides access to models. It returns a model’s location and not an artifact. In this scenario, for every participant, there is a RegisteredModel. For new training updates, the cluster runs a training job, pushes new weights to the underlying storage (e.g., Google Cloud Storage), and registers a new ModelVersion. The production container queries for the newest ModelVersion associated with the RegisteredModel that corresponds to the input participant. (2) Pre-generated containers, an offline training script running in a node pool that generates containers for every participant. Containers are pushed to the node pool and are kept in a cold state (not all active), and a node controller handles incoming requests and activates the relevant containers in the node pool. In this design, containers are kept alive for 30 min to handle repeat requests from the same participant and inference requests are then routed to that container.

Each of these two designs has its pros and cons. The Model Registry is useful when there are frequent training updates and the model is lightweight, which incurs less network overhead (e.g., individual-level models). On the other hand, the pre-generated container design is useful for large models with less frequent updates (i.e., cohort-level models). The ML cluster leverages distributed messaging for asynchronous communication across different microservices (i.e., a set of light-weight services that can scale independently).

#### MLflow

MLflow is open-source ML lifecycle management infrastructure. Its major relevant components include a tracking component to help data scientists monitor relevant metrics during training and a registry component where data science groups can register production models with versions. Crucially, these components are part of a larger collaboration hub hosted for the entire data science team, allowing the team to collaborate on identifying the best performing models while clearly versioning and managing which of those models are at what stage of deployment. This is important because this infrastructure ensures reproducibility—a deployed model’s full lineage from training to production is clearly preserved. Configuring MLflow on GCP, AWS, and Azure can be done following documentation—for research scale, the service is easy to deploy by connecting GCS as an artifact store and serving the server behind a reverse proxy. The service is cloud agnostic and can integrate with every cloud.

#### Back-end database

For the back-end database, some limitations are associated with the underlying computing frameworks. BigQuery and Athena, two commercial serverless and interactive query services, scale automatically based on their workloads. In contrast, Apache Presto, an open-source distributed query engine that supports much of the SQL analytics, utilizes Hadoop clusters, a different computing environment based on a distributed computing architecture. To configure Apache Presto, we used Google Dataproc^45^, a managed cloud service for running Apache Spark and Apache Hadoop. To achieve peak performance, for both serverless and non-serverless solutions, system engineers need to tune their solutions based on the number of requests and the size of the table, as well as the complexity of the queries as explained in the section “PHD is a scalable platform for data storage and analysis”.

#### Distributed messaging

To create a collection of autonomous services, PHD leverages microservices architecture and decouple its major components (e.g., SFTP Cluster, ML cluster) into smaller services that are independently developed, deployed, and maintained. Distributed messaging systems (e.g., GCP/AWS Pub/Sub, Apache Pulsar) enable lightweight communication across these services. The built-in durability and fault-tolerance of these messaging systems eliminate dropping messages and simplifies retries.

### Analysis

In the next subsections, we discuss the analysis details for Fig. 5 b, c, d, e. For the analysis details for Fig. 5f, refer to ref. ^30^.

#### Data cleaning and preprocessing

Raw wearable data were obtained in the second resolution for heart rate and sleep but in minute resolution for steps. Data were preprocessed on a per-individual basis, where we first aggregated all available data points for each individual transformed to 1-min-resolution. Fitbit devices provide sleep information across one of eight categories: asleep, awake, wake, light, deep, restless, REM, and unknown. Since this categorical mapping is obtained through algorithmic inference on the Fitbit device, we simplified the information and binarized the sleep information. A note providing details on the sleep mapping is included in Supplementary Table 2. In addition to sleep binarization, heart rate measurements were cleaned by excluding values below 20. Finally, data were smoothed using a rolling average with a 5 min window for heart rate and steps in conjunction with a rolling max operator for the binarized sleep information. Since subsequent analysis is based on summary statistics to avoid the introduction of imputation biases, no imputation was applied. Data aggregation and preprocessing resulted in a dataset covering 33 users.

#### Extraction of domain-knowledge-based features

Prior to feature extraction, wearables data were preprocessed on a per-day level as described in Methods subsection “Data cleaning and preprocessing”. Subsequently, for each day of wearables data (i.e., 24 h of data in 5-min resolution), 12 features were computed. Besides the average heart rate, total step count, and the number of sleep hours, additional features were computed by stratifying the observed heart rate measurements into three categories: sleeping heart rate, resting heart rate, and active heart rate. Sleeping heart rate measurements were defined as all heart rate measurements observed within a specific day along with a binary sleep indicator of 1, while resting heart rate was defined as all heart rate measurements observed in combination with a step count of 0. Similarly, active heart rate measurements comprised all heart rate measurements observed along with movement (i.e., a positive step count in the same time window). In addition, the difference between active and sleeping heart rate was computed. After heart rate stratification, the percentage of outliers was calculated by dividing the number of outliers within each feature by the total number of measurements for that respective feature. For this step, values exceeding 2 standard deviations of the corresponding variable distribution were considered outliers. Extraction of domain-knowledge-based features resulted in a vector of length 12 for each day of wearables data in the dataset. An overview of the calculated domain-knowledge-based features is provided in Supplementary Table 2.

#### Visualization of the feature space

Graphs were generated using the matplotlib python package version 3.1.3^46^. A lower dimensional embedding for visualization of the features space was generated using UMAP as implemented in the UMAP python package version 0.3.10^47^. Days with a total step count of 0 were excluded and features were log-transformed and standardized to zero-mean and unit variance. These steps resulted in a dataset of 33 individuals with 9512 days of data.

#### Logistic regression

Binary regularized logistic regression models were trained in a 10 × 5-fold cross-validation setting independently for classifying users’ sex (male vs. female) and insulin resistance (insulin resistant vs. insulin sensitive). Prior to the analysis, user data were preprocessed to extract per-day level features as described in Methods subsection “Data cleaning and preprocessing”. We excluded individuals without label information (i.e., information on sex or insulin resistance) and for both we excluded individuals with <14 days of data after preprocessing. We further restricted the analysis to the first 90 days (3 months) of data in order to match the wearables data to the clinical information, since measurements involving a clinical assessment were conducted at the baseline of the iPOP study^3,28^. Filtering and preprocessing resulted in datasets of 23 individuals with information on insulin resistance and 33 individuals with information on sex. For each individual, we subsequently calculated user-level summary statistics for each of the 12 domain-knowledge-based features comprising the mean, standard deviation, minimum, maximum and the 25%, 50%, and 75% quartiles, respectively. This resulted in a set of 84 features per user. Entering the cross-validation analysis, the data were randomly split into five non-overlapping partitions. For each of the splits, the classification model was trained on four of the partitions (training set) and evaluated on the fifth partition (test set). Specifically, the training set was normalized using the PowerTransform method^48^, and normalization parameters fitted on the train set were applied to normalize the test set. Subsequently, a L1-L2 regularized logistic regression (ratio of 0.5) was trained on the normalized train data. Each model’s predictions on the respective normalized test partition were combined to calculate the area under the receiver operating characteristic (AUROC) and the average precision score over the entire dataset. We repeated this random split and model evaluation procedure ten times and collected ten test-set AUROCs and average precision scores. All statistical analyses were performed in python 3.6 and the scikit-learn package^49^ version 0.22.1. Results were visualized using the matplotlib package^46^ version 3.1.3.

### Reporting summary

Further information on research design is available in the Nature Research Reporting Summary linked to this article.

## Supplementary information

Supplementary Information

Reporting Summary

## Data Availability

The wearables data analyzed in this study are publicly available at https://storage.googleapis.com/gbsc-gcp-project-ipop_public/PHD/PHD-paper-cohort-example-data.zip. The dataset for the COVID-19 detection study is publicly available at https://storage.googleapis.com/gbsc-gcp-project-ipop_public/COVID-19/COVID-19-Wearables.zip. Source data are provided with this paper.

## Source data

Source Data

## Code availability

We implemented the PHD platform in Python, Java, and Swift programming languages as a standalone software. The software is available at https://github.com/StanfordBioinformatics/personal-health-dashboard.
