# Supplementary material for: A scalable, secure, and interoperable platform for deep data-driven health management
Source: Nat Commun. 2021 Oct 1;12:5757. doi: 10.1038/s41467-021-26040-1 (PMC8486823; doi:10.1038/s41467-021-26040-1)
Supplement: Supplementary file 1 — Supplementary Information [file 41467_2021_26040_MOESM1_ESM.pdf]

Component	Cloud providers			HPC Cluster
	Google Cloud Platform	Amazon Web Services	Microsoft Azure	
Kuberenetes	Google Kubernetes Engine (GKE)	Amazon Elastic Kubernetes Service (Amazon EKS)	Azure Kubernetes Service (AKS)	Kubernetes (K8s)
Distributed Serverless Platform Executing Functions	Google Cloud Functions	AWS Lambda	Azure Functions	Apache OpenWhisk
SFTP	SFTPGo	SFTPGo	SFTPGo	SFTPGo
Back-end Database	Google BigQuery/Google Cloud Storage	Amazon Athena/Amazon S3	Azure Data Lake Analytics/Azure Blob Storage	Apache Presto/Hadoop Distributed File System (HDFS)
Terraform	✓	✓	✓	✓
MLflow	✓	✓	✓	✓

Supplementary Table 1. The platform is deployable on other cloud providers and HPC systems.

Number	Feature	Description
1	Heart rate	The average heart rate
2	Total step count	The total step count
3	Sleep hours	The number of hours spent asleep (see note below)
4	Resting heart rate	The average of all heart rate measurements observed with 0 steps
5	Sleeping heart rate	The average of all heart rate measurements observed while asleep (see note below)
6	Active heart rate	The average of all heart rate measurements observed with a step count > 0
7	Change active/sleeping heart rate	The difference of the mean active heart rate and the mean sleeping heart rate
8	Percentage of outliers heart rate	Measurements deviating more than two standard deviations from the distribution mean were treated as outliers
9	Percentage of outliers active heart rate	
10	Percentage of outliers resting heart rate	
11	Percentage of outliers sleeping heart rate	
12	Percentage of outliers in steps	
Note: Sleep was treated as binary condition, with the categories unknown, light, deep, restless, REM, asleep being treated as 1(= sleep) and the categories wake and awake being treated as 0 (= awake).		

Supplementary Table 2. For each iPOP participant, we generated 12 domain-knowledge-based features, such as the average heart rate, sleep, and activity-based heart rate stratification or total step counts from the Fitbit smartwatch data for each day.
