# Supplementary material for: A scalable, secure, and interoperable platform for deep data-driven health management
Source: Nat Commun. 2021 Oct 1;12:5757. doi: 10.1038/s41467-021-26040-1 (PMC8486823; doi:10.1038/s41467-021-26040-1)
Supplement: Supplementary file 2 — Reporting Summary [file 41467_2021_26040_MOESM2_ESM.pdf]

nature research

Corresponding author(s): Xiao Li
Michael Snyder

Last updated by author(s): Aug 9, 2021

Reporting Summary

Nature Research wishes to improve the reproducibility of the work that we publish. This form provides structure for consistency and transparency in reporting. For further information on Nature Research policies, see our Editorial Policies and the Editorial Policy Checklist.

Statistics

For all statistical analyses, confirm that the following items are present in the figure legend, table legend, main text, or Methods section.

n/a	Confirmed	
☐	☒	The exact sample size (n) for each experimental group/condition, given as a discrete number and unit of measurement
☐	☒	A statement on whether measurements were taken from distinct samples or whether the same sample was measured repeatedly
☒	☐	The statistical test(s) used AND whether they are one- or two-sided<br>*Only common tests should be described solely by name; describe more complex techniques in the Methods section.*
☐	☒	A description of all covariates tested
☐	☒	A description of any assumptions or corrections, such as tests of normality and adjustment for multiple comparisons
☐	☒	A full description of the statistical parameters including central tendency (e.g. means) or other basic estimates (e.g. regression coefficient) AND variation (e.g. standard deviation) or associated estimates of uncertainty (e.g. confidence intervals)
☒	☐	For null hypothesis testing, the test statistic (e.g. F, t, r) with confidence intervals, effect sizes, degrees of freedom and P value noted<br>*Give P values as exact values whenever suitable.*
☒	☐	For Bayesian analysis, information on the choice of priors and Markov chain Monte Carlo settings
☒	☐	For hierarchical and complex designs, identification of the appropriate level for tests and full reporting of outcomes
☒	☐	Estimates of effect sizes (e.g. Cohen's d, Pearson's r), indicating how they were calculated

Our web collection on statistics for biologists contains articles on many of the points above.

Software and code

Policy information about availability of computer code

Data collection	Mobile development: Swift 4 and Java - Xcode (12.4) and Android Studio (4.1.2)<br>Cloud Computing services: GCP BigQuery (1.22.0), GCP Storage (1.22.0), GCP Cloud Functions, Terraform (0.12.29), fail2ban (0.10.4), Kubernetes (1.18), SFTPGo (2.0)
Data analysis	All statistical analyses were performed in python 3.6 and the scikit-learn package version 0.22.1. Results were visualized using the matplotlib package version 3.1.3. GitHub: https://github.com/StanfordBioinformatics/personal-health-dashboard

For manuscripts utilizing custom algorithms or software that are central to the research but not yet described in published literature, software must be made available to editors and reviewers. We strongly encourage code deposition in a community repository (e.g. GitHub). See the Nature Research guidelines for submitting code & software for further information.

Data

Policy information about availability of data

All manuscripts must include a data availability statement. This statement should provide the following information, where applicable:

- Accession codes, unique identifiers, or web links for publicly available datasets
- A list of figures that have associated raw data
- A description of any restrictions on data availability

The wearables data analyzed in this study are publicly available at:
https://storage.googleapis.com/gbsc-gcp-project-ipop_public/PHD/PHD-paper-cohort-example-data.zip
The dataset for the COVID-19 detection study are publicly available at:
https://storage.googleapis.com/gbsc-gcp-project-ipop_public/COVID-19/COVID-19-Wearables.zip

Field-specific reporting

Please select the one below that is the best fit for your research. If you are not sure, read the appropriate sections before making your selection.

- [x] Life sciences
- [ ] Behavioural & social sciences
- [ ] Ecological, evolutionary & environmental sciences

For a reference copy of the document with all sections, see nature.com/documents/nr-reporting-summary-flat.pdf

Life sciences study design

All studies must disclose on these points even when the disclosure is negative.

Sample size	Sample size for the iPOP project was calculated based on findings of levels of autocorrelation in multiomics data and the number of timepoints needed per participant to reconstruct the time series with < 1% uncertainty. Additionally, our observational study is different from traditional cross-section study in two aspects: 1) We sample more than one time longitudinally in each of the participants; 2) We focus on naturally or spontaneously occurring physiological states. For group-wise comparison, we examined on those had at least 30 individuals or events per group (IR vs IS, Infection vs healthy, Immunization vs infection etc) and used statistical analyses to take the sample size into account. The sample size is confirmed sufficient by using a stringent significant threshold in downstream statistics. For the COVID19 study, in terms of sample size, the crucial aspect was to ensure maximal high-frequency sampling within an individual before and around the time of COVID-19 infection. We recruited as broadly as possible, and tried to maximize the number of individuals, but were limited by the infection rates during the recruitment period of the study.
Data exclusions	For the COVID19 study, we only released data for participants that we had enough wearable data and hence a complete analysis.<br>We further restricted the logistic regression analysis to the first 90 days (3 months) of data in order to match the wearables data to the clinical information, since measurements involving a clinical assessment were conducted at the baseline of the iPOP study.
Replication	This was an observational study in which we did not perform experiments.
Randomization	This is not a randomized human study.
Blinding	Each participant was labeled with a randomized ID whose annotation and clinical status were kept blinded during data collection and analyses.

Reporting for specific materials, systems and methods

We require information from authors about some types of materials, experimental systems and methods used in many studies. Here, indicate whether each material, system or method listed is relevant to your study. If you are not sure if a list item applies to your research, read the appropriate section before selecting a response.

Materials & experimental systems

n/a	Involved in the study	
x		Antibodies
x		Eukaryotic cell lines
x		Palaeontology and archaeology
x		Animals and other organisms
	x	Human research participants
x		Clinical data
x		Dual use research of concern

Methods

n/a	Involved in the study	
x		ChIP-seq
x		Flow cytometry
x		MRI-based neuroimaging

Human research participants

Policy information about studies involving human research participants

Population characteristics	The detailed population characteristics of the COVID-19 study were reported in Zhou et. al. 2019; 55 participants in this study were female and 51 male, with ages ranging from 25 to 75 yrs old and BMI 25-40 kg/m2. The detailed population characteristics of the COVID-19 study were reported in Mishra et. al. 2020. The mean age of the participants at time of enrollment was 44 (range, 18–88); 55.3% were women. However, out of the 32 individuals analyzed, 25 (78.1%) were women. The self-reported ethnic distribution of the full cohort was 74.9% European, 3.9% East Asian, 2.9% African American, 19.2% Mixed/Other/Undeclared. The most common self-reported health conditions at entry were respiratory lung disease, high blood pressure, high cholesterol, and allergy/immune disease.
Recruitment	The iPOP participants were recruited via placement of advertisements in local newspapers and radio stations seeking “prediabetic volunteers” at risk for Type 2 diabetes for longitudinal multi-omic study. Screening in the CTRU entailed history and physical, anthropometric measurements, and fasting blood tests for exclusions including presence of anemia defined as hematocrit < 30, renal disease defined as creatinine > 1.5, history of any cardiovascular, malignancy, chronic inflammatory, psychiatric disease, and history of any bariatric surgery or liposuction. The COVID-19 participants were recruited by social media, word of mouth, and through Stanford Healthcare. Our inclusion criteria screened for people who either had a COVID-19 infection or were at high risk through living situation or employment, and were wearing fitness trackers. Since social media was one of the recruitment methods used, there could be a bias towards people who use social media. The use of wearable devices likely biases our study cohort towards individuals of higher socioeconomic strata, who are more likely to be able to afford the devices. It is also possible that individuals who own wearables devices are more interested in using wearable devices to monitor both activity and health. However, we do not think that the results of our algorithm are affected by interest in wearables devices.
Ethics oversight	Stanford University Institutional Review Board (IRB 23602, IRB 55577, IRB 57022, IRB 34907)

Note that full information on the approval of the study protocol must also be provided in the manuscript.
